# Revisiting the loading dose of amikacin for patients with severe sepsis and septic shock

**DOI:** 10.1186/cc8945

**Published:** 2010-04-06

**Authors:** Fabio Silvio Taccone, Pierre-François Laterre, Herbert Spapen, Thierry Dugernier, Isabelle Delattre, Brice Layeux, Daniel De Backer, Xavier Wittebole, Pierre Wallemacq, Jean-Louis Vincent, Frédérique Jacobs

**Affiliations:** 1 Department of Intensive Care, Erasme Hospital, Université Libre de Bruxelles, Route de Lennik 808, 1070 Bruxelles, Belgium; 2 Department of Intensive Care, Cliniques Universitaires St-Luc Avenue Hippocrate 10, 1200 Bruxelles, Belgium; 3 Department of Intensive Care, Universitair Ziekenhuis Brussel, Laerbeeklaan 101, 1090 Bruxelles, Belgium; 4 Department of Intensive Care, St-Pierre Hospital, Avenue Reine Fabiola 9, 1340 Ottignies, Belgium; 5 Department of Clinical Biochemistry and Pharmacokinetics, Cliniques Universitaires, St-Luc Avenue Hippocrate 10, 1200 Bruxelles, Belgium; 6 Department of Infectious Diseases, Erasme Hospital, Université Libre de Bruxelles, Route de Lennik 808, 1070 Bruxelles, Belgium

## Abstract

**Introduction:**

It has been proposed that doses of amikacin of >15 mg/kg should be used in conditions associated with an increased volume of distribution (V_d_), such as severe sepsis and septic shock. The primary aim of this study was to determine whether 25 mg/kg (total body weight) of amikacin is an adequate loading dose for these patients.

**Methods:**

This was an open, prospective, multicenter study in four Belgian intensive care units (ICUs). All consecutive patients with a diagnosis of severe sepsis or septic shock, in whom amikacin treatment was indicated, were included in the study.

**Results:**

In 74 patients, serum samples were collected before (t = 0 h) and 1 hour (peak), 1 hour 30 minutes, 4 hours 30 minutes, 8 hours, and 24 hours after the first dose of amikacin. Blood amikacin levels were measured by using a validated fluorescence polarization immunoassay method, and an open two-compartment model with first-order elimination was fitted to concentrations-versus-time data for amikacin (WinNonlin). In 52 (70%) patients, peak serum concentrations were >64 μg/ml, which corresponds to 8 times the clinical minimal inhibitory concentration (MIC) breakpoints defined by EUCAST for Enterobacteriaceae and *Pseudomonas aeruginosa *(S<8, R>16 μg/ml). V_d _was 0.41 (0.29 to 0.51) L/kg; elimination half-life, 4.6 (3.2 to 7.8) hours; and total clearance, 1.98 (1.28 to 3.54) ml/min/kg. No correlation was found between the amikacin peak and any clinical or hemodynamic variable.

**Conclusions:**

As patients with severe sepsis and septic shock have an increased V_d_, a first dose of ≥ 25 mg/kg (total body weight) of amikacin is required to reach therapeutic peak concentrations. However, even with this higher amikacin dose, the peak concentration remained below therapeutic target levels in about one third of these patients. Optimizing aminoglycoside therapy should be achieved by tight serum-concentration monitoring because of the wide interindividual variability of pharmacokinetic abnormalities.

## Introduction

Severe sepsis and septic shock are major causes of morbidity and mortality in intensive care units (ICUs) [1]. Early and appropriate infection control is a priority in the management of sepsis and requires adequate early administration of effective antibiotics with a dosing strategy able to achieve therapeutic concentrations at the site of infection [2,3].

Aminoglycosides are often given as part of empiric therapy for severe sepsis and septic shock, especially if *Pseudomonas aeruginosa *infection is suspected. Their use is further supported by the emergence of multidrug-resistant bacteria and the lack of new drugs active against these microorganisms [4]. Amikacin is a highly potent, broad-spectrum aminoglycoside that is usually given in association with β-lactams for the treatment of severe gram-negative infections [5]. Meta-analyses have shown limited and conflicting benefits from this combination therapy [6,7]. However, the paucity of trials including patients with severe sepsis and septic shock precludes any recommendations in this setting, and the different amikacin doses and regimens used may have led to inadequate drug concentrations.

In clinical practice, the ratio between the peak and the minimum inhibitory concentration (MIC) of the causative gram-negative pathogen (peak/MIC) is considered to be the parameter that best characterizes the *in vivo *exposure of the pathogen to serum aminoglycoside concentrations [8,9]. Optimal antibacterial activity is achieved when the peak is 8 to 10 times greater than the MIC [10-12]. Despite a large variance in MIC values for different bacteria, therapy should usually target problematic pathogens in ICU patients, such as Enterobacteriaceae and *Pseudomonas aeruginosa*. The clinical MIC breakpoint for these pathogens is 8 μg/ml [13], indicating that to optimize the antibacterial activity of amikacin, peak drug concentrations should reach ≥ 64 μg/ml. This strategy would allow these "difficult-to-treat" pathogens to be exposed to bactericidal drug concentrations, even when treatment is initiated empirically without any knowledge of specific MICs.

Although aminoglycoside pharmacokinetics (PKs) have been already described for the treatment of ICU infections, studies on optimal regimens in sepsis patients had several limiting factors [14-18]. Few prospective data are available regarding which aminoglycoside dose should be used to optimize aminoglycoside concentrations in ICU patients with severe sepsis and septic shock with multiple organ dysfunction. Also, no data are available about the impact of using ideal body weight (IBW) or total body weight (TBW) on the achievement of optimal peak amikacin concentrations in ICU patients.

The primary aim of this study was to validate a higher dosing regimen for amikacin in patients with severe sepsis and septic shock. We also evaluated the impact of body weight, and specifically of an IBW-compared with a TBW-based regimen, on the optimization of peak amikacin concentrations. Finally, we evaluated whether particular clinical or hemodynamic parameters influenced amikacin PK and propose new recommendations for the loading dose of amikacin in this critically ill population.

## Materials and methods

### Study design, patients, and antibiotic treatment

This was an open, prospective, multicenter, noncomparative study performed in four polyvalent ICUs from four Belgian hospitals between January 2005 and June 2006. The study protocol was approved by the Ethics Committees of the different hospitals. Written informed consent was obtained from each patient or his or her legal guardian. Patients with a diagnosis of severe sepsis or septic shock according to standard criteria [19], in whom amikacin treatment was indicated, were consecutively enrolled in the study. The aminoglycoside was given in combination with a broad-spectrum β-lactam (ceftazidime, cefepime, piperacillin-tazobactam, or meropenem), according to local clinical practice. Exclusion criteria were younger than 18 years of age, pregnancy, burns or cystic fibrosis (because of increased V_d_), neuromuscular disease, body mass index (BMI) >40 kg/m^2^, chronic renal failure requiring dialysis, amikacin treatment in the previous 2 weeks, and known allergy to aminoglycosides. No patient was included more than once. The study period was limited to the first 24 hours of treatment.

All patients included in the study received a loading dose of 25 mg/kg of amikacin based on TBW; this regimen was defined for an expected mean V_d _of 0.4 L/kg and a target peak of 64 μg/ml [17,20,21]. Doses were rounded off at multiples of 125 mg. The drug was administered over a 30-min period by using an infusion pump, and the tubing was flushed with 0.9% sodium chloride after the dose was administered. Blood samples for drug assays were taken immediately before administration (0 h) and 1 h, 1 h 30, 4 h 30, 8 h, and 24 h thereafter. These blood-sampling time points are supposed to belong to the elimination phase of the drug. The exact time of sampling was recorded. Blood was collected in a 5-ml plain tube (without anticoagulant). When a clot had completely formed (15 to 30 min), the sample was centrifuged at 4°C, and the serum was stored at - 80°C until analysis.

### Analytic method for amikacin

Amikacin concentrations were quantified at the end of the study in a central reference laboratory (St-Luc Hospital) by using a validated fluorescence polarization immunoassay with the TDx analyzer (Abbott Laboratories, Abbott Park, IL, USA). Routine daily quality controls (5, 15, and 30 μg/ml) and calibrators (3, 10, 20, 35, and 50 μg/ml) were provided by Abbott Laboratories. No sample preparation was required for the assay. According to the manufacturer, the limit of quantification (LOQ) is 0.8 μg/ml.

### PK analysis

Serum amikacin concentrations were analyzed by using WinNonlin Pharsight Professional Software Version 5.0.1 (Pharsight Corporation, Mountain View, CA, USA). One-compartment and two-compartment open models with first-order elimination were compared to fit amikacin PKs data. A two-compartment model was selected as the best to fit the Concentration-versus-Time data for amikacin (data not shown). The following pharmacokinetic variables were calculated for each patient: the volume of distribution in the central (V_d1_) and in the peripheral (V_d2_) compartments, total volume of distribution (V_ss_), total clearance (CL), elimination half-life (t_1/2_), area under the curve (AUC) during the 24 hours, C_max _(maximal concentration calculated by extrapolation of the distribution phase,) and C_min _(concentration 24 h after the start of infusion).

### PK end points

Amikacin levels measured 1 hour (= peak) after the onset of perfusion [8-11,15,21] were considered the target concentrations. Optimal peak was considered as >64 μg/ml. The potential toxicity threshold of the drug was determined by a C_min _>5 μg/ml [15,16]. However, no evaluation of changes in renal function was performed after the first day of therapy.

### Weight estimation

Body weight was considered on the day of amikacin administration. TBW was taken from medical files for patients admitted from the floor or the operating room; in case of admission through the Emergency Department, institutional databases with recent hospitalizations were used. TBW was also asked directly of the patients, whenever possible. TBW was estimated by doctors and nurses in 10 patients. IBW was calculated according to Devine's formula [22]. Corrected body weight (DW) for patients with BMI <20 and >28 kg/m^2 ^was calculated according to previous recommendations [23-25]: for BMI >28 kg/m^2^, DW = 0.4 × (TBW - IBW) + IBW; for BMI <20 kg/m^2^, DW = 1.13 × IBW. By using the same PK model obtained with a TBW-based regimen, simulations of individual PK profiles were performed to assess the effect of IBW- and DW-based regimens on peak and C_min _concentrations.

### Data collection

Demographic data, comorbidities, and admission diagnoses were collected in all patients. Disease severity was characterized by the Acute Physiology and Chronic Health Evaluation (APACHE) II score [26]. Organ dysfunction was assessed by using the Sequential Organ Failure Assessment (SOFA) score [27] on the first day of antibiotic treatment. Positive microbiologic cultures were recorded. Site of infection was defined according to the Centers for Disease Control definitions [28]. Biologic data, including coagulation parameters, complete blood count, electrolyte, urea, creatinine, bilirubin, total protein and albumin concentrations, myocardial and liver enzymes, and C-reactive protein (CRP) concentrations, were recorded at inclusion and at 24 hours. Creatinine clearance (CrCl) was estimated with the Cockcroft and Gault equation by using TBW [29]. Renal dysfunction was considered when CrCl was <50 ml/min [30]. Acute renal failure was defined as a renal SOFA score >2 (creatinine > 3.0 mg/dl and/or urine output <500 ml/day) and/or need for renal replacement therapy [27]. Other recorded parameters were the use of adrenergic drugs, mechanical ventilation, renal support, 24-hour fluid balance, length of ICU stay, ICU mortality, and cause of death. Hemodynamic and blood-gas analysis data were collected at baseline and 8 and 24 hours after the start of the protocol.

### Statistical analysis

Statistical analyses were performed by using the SPSS 13.0 for the Windows NT software package (SPSS Inc. 2004). Descriptive statistics were computed for all study variables. A Kolmogorov-Smirnov test was used, and histograms and normal-quantile plots were examined to verify the normality of distribution of continuous variables. Discrete variables were expressed as counts (percentage), and continuous variables, as mean ± SD or median [25th-75th percentiles]. Demographics and clinical differences between study groups were assessed by using a χ^2^, Fisher's Exact test, Student's *t *test, or Mann-Whitney *U *test, as appropriate. The Pearson's (*r*) correlation coefficient was used to determine linear correlation. Association between variables was tested by simple regression analysis and coefficient of determination (R^2^) in the case of nonlinear correlation. An univariate analysis followed by a multivariate stepwise linear-regression analysis, including all the collected variables, was also performed to predict the amikacin peak. A value of *P *< 0.05 was considered to be statistically significant.

## Results

### Characteristics of patients

We enrolled 74 patients (Table 1). The median APACHE II score was 21, and the median SOFA score on admission was 8. Fifty-six (76%) patients were treated with mechanical ventilation, and 20 (27%) patients had acute renal failure. Overall ICU mortality was 36%; 22 of 27 deaths were attributed to sepsis or related multiple organ failure. Most infections were respiratory or abdominal and were microbiologically documented in 50 (68%) patients. Blood cultures were positive in 29 (39%) patients. Forty-three (58%) cases of sepsis were secondary to gram-negative bacilli, with 28 infections due to difficult-to-treat pathogens (*P. aeruginosa *(n = 15); *Enterobacter *spp. (n = 8); *Serratia marcescens *(n = 2); *Citrobacter freundii*, *Hafnia alvei*, or *Morganella morganii *(each n = 1)).

**Table 1:** Patient characteristics, hemodynamic and biologic data on admission, and fluid balance during the first 24 hours

Variables	Values
Age (years)	63 ± 13
Sex (Male/Female)	50/24
Body Mass Index	24.7 ± 4.6
APACHE II score	21 (16-26)
SOFA score on admission	8 (5-11)
Medical/Surgical	50/24
COPD	14 (19%)
Diabetes	17 (23%)
Heart disease	39 (53%)
Chronic renal insufficiency	7 (9%)
Liver cirrhosis	11 (15%)
Immunosuppressive drugs	25 (34%)
Malignancy	24 (32%)
	
Community/hospital-acquired infections	22/52
Severe sepsis/septic shock	17/56
Mechanical ventilation	56 (76%)
Acute renal failure	20 (27%)
Vasopressor agents	56 (76%)
ICU stay (days)	14 (5-25)
Overall ICU mortality	27 (37%)
Mean arterial pressure (mmHg)	70 ± 14
Central venous pressure (mmHg)	10 ± 5
pH	7.41 ± 0.12
PaO_2_/FiO_2 _ratio	166 [112-227]
PaCO_2 _(mmHg)	39 ± 12
Lactate (mEq/L)	2.7 [1.7-3.9]
	
White blood cells (/mm^3^)	11400 [8600-20400]
Hematocrit (%)	30.4 ± 7.6
Platelets (10^3^/mm^3^)	175 [86-292]
Bilirubin (mg/dl)	0.9 [0.5-2.0]
Creatinine (mg/dl)	1.7 [1.1-3.1]
C-reactive protein (mg/dl)	17 [10-26]
Creatinine clearance (ml/min)	54 [33-86]
	
Fluid balance (ml/24 h)	2650 ± 2121
Fluids IN (ml/24 h)	4594 ± 1892
Fluids OUT (ml/24 h)	1944 ± 1621

Data are expressed as counts (percentage), median (IQR), or mean ± SD. APACHE, Acute Physiology and Chronic Health Evaluation; COPD, chronic obstructive pulmonary disease; ICU, intensive care unit; SOFA, Sequential Organ Failure Assessment.

### Pharmacokinetic data

**Figure 1:**
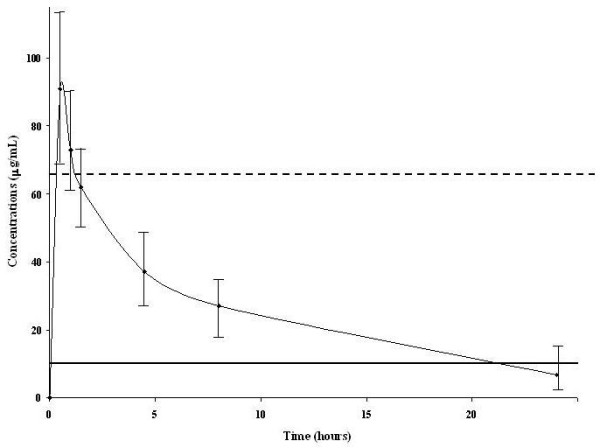
**Pharmacokinetic profile of amikacin**. Dashed line, peak of 64 μg/ml corresponding to 8 times the clinical breakpoint of the minimal inhibitory concentration (MIC = 8 μg/ml, solid line) for gram-negative bacteria.

**Figure 2:**
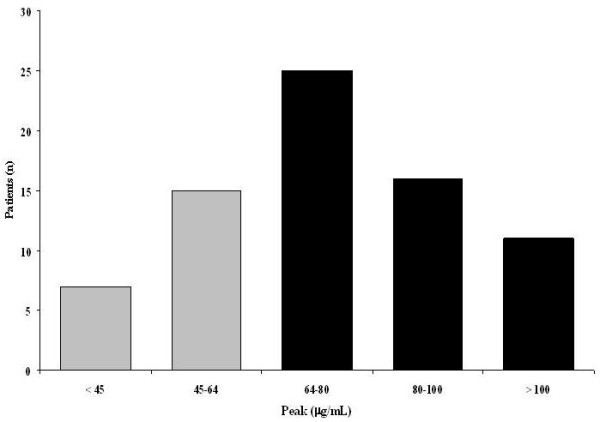
**Distribution of peak concentrations**. Black bars, peak >64 μg/ml; gray bars, peak <64 μg/ml.

The median amikacin dose was 1,750 mg (range, 1,125 to 3,000 mg). Main PK parameters for amikacin were V_ss _0.41 [0.29 to 0.51] L/kg, t_1/2 _4.6 [3.2 to 7.8] hours, and CL 1.98 [1.28-3.54] ml/min/kg (Table 2). Median serum concentrations of amikacin were 0, 72.7 (61.7 to 90.2), 61.5 (48.5 to 73.1), 37.3 (27.7 to 46.5), 26.7 (16.4 to 33.8), and 6.7 (2.1 to 15.4) μg/ml at 0 hours, 1 hour (peak), 1 hour 30 minutes, 4 hours 30 minutes, 8 hours, and 24 hours, respectively (Figure 1). Peak serum concentrations were >64 μg/ml in 52 (70%) patients (Figure 2). For a target MIC of 8 μg/ml, the peak/MIC ratio was 9.6 ± 3.5. With this regimen, peak/MIC >8 would have been reached in all patients for an MIC of ≤ 4 μg/ml.

**Table 2:** Amikacin pharmacokinetics in patients with or without renal dysfunction

	All (n = 74)	CrCl <50 ml/min (n = 38)	CrCl >50 ml/min (n = 36)
V_d1 _(central) (L)	0.23 (0.18-0.28)	0.23 (0.19-0.35)	0.22 (0.17-0.26)
V_d2 _(peripheral) (L)	0.18 (0.11-0.23)	0.19 (0.12-0.29)	0.18 (0.11-0.23)
V_ss _(L/kg)	0.41 (0.29-0.51)	0.42 (0.31-0.54)	0.40 (0.28-0.49)
t_1/2 _(hours)	4.6 (3.2-7.8)	7.6 (4.6-13.2)	3.3 (2.5-4.6)^a^
CL (ml/min/kg)	1.98 (1.28-3.54)	1.29 (0.84-2.01)	3.49 (1.94-5.04)^a^
AUC (mg × h/ml)	798 (478-1285)	1,177 (833-1961)	466 (368-763)^a^
C_max _(μg/ml)	91.7 (73.1-112.8)	91.0 (70.6-112.8)	92.3 (74.1-109.4)
Peak (μg/ml)	72.7 (61.7-90.2)	71.5 (57.3-86.3)	75.7 (63.1-92.9)
C_min _(μg/ml)	6.7 (2.1-15.4)	15.4 (8.0-21.4)	2.6 (1.3-6.0)^a^

Data are expressed as median (IQR). ^a ^*p *< 0.001. AUC, area under the curve; t_1/2_, elimination half-time; CL, clearance; CrCl, creatinine clearance; V_d_, volume of distribution; V_ss_, total volume of distribution.

Thirty-nine (52%) patients had a C_min _>5 μg/ml. C_min _(R^2 ^= 0.51, *P *< 0.01) and CL (R^2 ^= 0.41; *P *<0.01), but not peak concentration, were correlated with CrCl at inclusion. Moreover, patients with renal dysfunction had higher amikacin levels at 4 hours 30 minutes (43.9 ± 11.5 μg/ml vs. 31.8 ± 14.4 μg/ml; *P *< 0.001) and 24 h (15.4 (8.0 to 21.4) μg/ml vs. 2.6 (1.3 to 6.0) μg/ml; *P *< 0.001), as well as lower CL and increased t_1/2 _and AUC, than had patients with normal renal function. However, the V_ss _and peak concentrations were similar (Table 2).

### Impact of weight on peak amikacin concentrations

Adequate peak concentrations were achieved in six (54%) of 11 patients with a BMI <20, 23 (64%) of 36 with a BMI of 20 to 25, 15 (83%) of 18 with a BMI from 25 to 30, and eight (89%) of nine with a BMI >30. Simulation with doses calculated by using the IBW showed that only 35 (47%) patients would have reached a peak >64 μg/ml (*P *< 0.01 compared with a regimen established by using TBW). The use of DW compared with TBW for dose calculation had no effect in terms of adequate peak concentrations (Table 3). When the amikacin dose was calculated by using DW, a dose of 28 mg/kg would have been necessary for 70% of patients to have achieved the desired peak concentration.

**Table 3:** Differences in numbers of patients achieving optimal peak or high C_min _concentrations

Regimen	Peak >64 μg/ml n (%)	**C**_min _**>5 μg/ml** n (%)
15 mg/kg TBW	7 (9)	29 (39)
25 mg/kg TBW	50 (72)	39 (52)
30 mg/kg TBW	59 (79)	43 (58)
25 mg/kg IBW	35 (47)	39 (52)
25 mg/kg DW	42 (56)	39 (52)

Doses were calculated by using total body weight (TBW), ideal body weight (IBW), or IBW with correction factors (DW) for extreme body mass indexes.

### Simulations with other regimens

By using a simulated amikacin dose of 15 mg/kg, we observed that only seven (9%) patients would have reached a peak concentration >64 μg/ml (*P *< 0.001 vs. 25 mg/kg TBW dose), whereas for a 30-mg/kg regimen, 80% of patients would have achieved the optimal peak concentration (Table 3). Interestingly, the proportion of patients with a C_min _>5 μg/ml would have been similar to the 15 and 25 mg/kg regimens (39% vs. 52%; *P *= 0.1).

### Correlation with clinical variables

The peak of amikacin was not correlated with or predicted by any biologic, hemodynamic, or clinical variable, including age, mechanical ventilation, APACHE II or SOFA score at admission, presence of shock, maximal dose of vasopressor agents, fluid balance, or renal failure. Patients with a peak concentration >64 μg/ml had similar characteristics to patients with peak concentrations <64 μg/ml (Table 4).

**Table 4:** Differences between patients with optimal (>64 μg/ml) and inadequate (<64 μg/ml) peak amikacin concentrations

	Peak >64 (n = 52)	Peak <64 (n = 22)
Male	32 (62)	18 (81)
Nosocomial infections	37 (71)	15 (68)
Medical admission	37 (71)	13 (59)
Positive blood cultures	18 (35)	11 (50)
Shock/vasopressors	40 (76)	16 (72)
Mechanical ventilation	37 (71)	19 (86)
Age (years)	62 ± 14	64 ± 13
APACHE II score	20 (16-25)	23 (16-28)
SOFA score	8 (5-10)	9 (5-11)
Highest lactate (mmol/L)	3.1 (1.5-4.8)	2.8 (1.3-5.2)
Creatinine (mg/ml)	1.6 ± 1.1	1.7 ± 1.3
Albumin (g/dl)	2.4 ± 0.8	2.4 ± 0.8
Fluid balance (ml/24 h)	2,589 ± 2,153	2,793 ± 2,085

Data are expressed as median (IQR), mean (± SD), or counts (percentage). APACHE, Acute Physiology and Chronic Health Evaluation; SOFA, Sequential Organ Failure Assessment.

## Discussion

This is the first study in which a higher dose of amikacin was prospectively validated in sepsis patients after a PK analysis. We showed that, because of PK alterations, a loading dose of ≥ 25 mg/kg of amikacin is necessary to achieve therapeutic peak concentrations in patients with severe sepsis or septic shock. Antimicrobials PKs in ICU patients are significantly different from those in healthy volunteers or less severely ill patients [14,31,32]. Increased cardiac index and interstitial fluid shifts in sepsis result in a larger volume of distribution (V_d_), which may reduce plasma antibiotic levels [33]. Decreased protein binding can result in higher free-drug concentrations, and organ dysfunction may decrease drug metabolism and clearance [33]. Finally, infections, especially when acquired in the ICU, are often caused by more-resistant pathogens [34]. For aminoglycosides, peak concentration is determined by the administered dose and by the V_d _[30]. The V_d _of amikacin is between 0.2 and 0.3 L/kg in healthy volunteers and in patients with mild infections [12,20,35]. In our study, the median V_d _was 0.41 L/kg, corresponding to a >60% increase when compared with normal ranges. These results confirm data from previous studies. In 200 adult and pediatric ICU patients with severe gram-negative infections, the V_d _of amikacin varied from 0.17 to 0.98 L/kg, with a mean of 0.37 L/Kg [16]. A mean V_d _of 0.47 L/kg was reported in 30 ICU patients [17]. In patients with postoperative septic shock, the V_d _was 0.41 ± 0.08 L/kg, a significantly higher value than that in controls (0.25 ± 0.01 L/kg) [36]. The variability of V_d _in sepsis patients is probably multifactorial and depends on the degree of inflammation, vascular permeability, and fluid extravasation [12,32,37]. Doses of 15 and 20 mg/kg produced means of 33.5 ± 14.8 and 33.8 ± 4.7 μg/ml, respectively, in adult ICU patients [15,16]. However, the peak obtained with these regimens was largely below the desired concentration of 64 μg/ml, suggesting that higher doses of amikacin should be administered to achieve optimal peak levels. Moreover, previous studies on amikacin dose in ICU patients had limited patient samples [17,36,38], were retrospective [10,39], or had exclusion criteria, such as septic shock [15], APACHE II score >35 [40], liver cirrhosis [17], or acute renal failure [15-17,40], making it difficult to extrapolate the results to a general septic ICU population.

Our study is the first to provide data on sepsis patients with several comorbidities, high disease severity, and multiple organ dysfunctions, with an ICU mortality rate of nearly 40%. This cohort of 74 patients was relatively large and representative of a typical ICU population. Most of the infections were secondary to gram-negative infections, with 20% being caused by difficult-to-treat bacteria known to be associated with high mortality rates [41]. This represents the population in which aminoglycoside treatment could be recommended [6].

Assuming a three- to fourfold factor for converting doses of amikacin to gentamicin and tobramycin, it has been suggested that higher doses should be used for these two aminoglycosides in patients with septic shock [18,42]. However, a dose >7 mg/kg has not been prospectively validated for these drugs. Our data demonstrate that, with 25 mg/kg of amikacin, the target peak concentration (>64 μg/ml) was achieved in 70% of patients. An even higher dose may be necessary in some patients for whom the peak concentration remains below the desired level. Simulation with a standard regimen (15 mg/kg) of amikacin resulted in insufficient peak concentrations in >90% of patients, confirming the need to increase amikacin doses to ensure that adequate peak levels are achieved in sepsis patients.

A relation between the intensity of the septic process and the expansion of the V_d _can be assumed. Marik *et al*. [16,43] and Lugo-Goytia *et al*. [39] demonstrated an association between sepsis severity, estimated by the APACHE II score, and aminoglycoside V_d_. V_d _was also reported to be correlated with oxygen extraction ratio, serum albumin levels, and adrenergic support in another study [17]. We did not find any relation between V_d _and any demographic, clinical, hemodynamic, or biologic variable. Our population was analyzed in the early phase of the septic process, and this may explain the difference from previous studies, which were conducted in the steady state. The considerable interindividual variability observed in critically ill patients may also limit the *a priori *prediction of PK abnormalities and the optimal dose that should be administered to sepsis patients. Optimizing aminoglycoside therapy should, therefore, be achieved by tight serum-concentration monitoring (peak and trough) and rapid dose adjustment [44] according to pathogen MIC. This strategy requires pathogen MIC measurement and a C_min _<5 μg/ml to optimize the subsequent doses and to avoid drug accumulation.

Physiologic alterations associated with increased BMI affect the aminoglycoside PK. This is due to the variable penetration of these drugs into adipose tissue. Previous studies have validated dosing weight correction factors to normalize predictions of V_ss _in morbidly obese subjects [23] as well as in overweight/underweight patients [24] in a non-ICU population. Also, IBW seems to fit the pharmacokinetics of these antimicrobials better than the total body weight (TBW) to calculate the aminoglycoside regimen [45,46]; however, some uncertainty exists in this area [47]. Our results suggest that using a DW-based regimen could result in a relative underdosing of aminoglycoside in critically ill sepsis patients when compared with a TBW-based regimen. Thus, if using IBW, a loading dose even higher than 25 mg/kg should be considered in this patient population to obtain adequate amikacin peak concentrations. Importantly, a higher dosage should be considered also in patients with a BMI <20 to avoid underdosage. We excluded morbidly obese patients (BMI >40), so that we cannot comment on this particular population.

Our study has some limitations. First, we evaluated the PK profile of amikacin only during the first 24 hours of administration, and thus cannot make any statement with regard to subsequent doses. The V_d _may decrease during therapy when capillary leakage subsides and sepsis resolves [48]. In these circumstances, amikacin doses <25 mg/kg may be sufficient to achieve therapeutic concentrations.

Second, a control group of patients without sepsis was not included. However, it would have been unethical to expose nonseptic patients, even with increased V_d_, such as in trauma or cardiac surgery [5,49], to a higher dose of a potentially toxic antibiotic drug.

Third, we did not evaluate the evolution of renal function in our population, with amikacin concentrations exceeding the toxicity limit at 24 hours in >50% of patients. However, targeting peak amikacin concentrations >60 μg/ml resulted in the same incidence of nephrotoxicity compared with conventional treatment [40], as long as individualized pharmacokinetic dosing of aminoglycoside was performed to allow a necessary drug-free period. The CrCl was estimated by using the Crockroft and Gault formula; however, this may overestimate CrCl because immobility and reduced muscle mass in ICU patients and a more accurate assessment of renal function should have been based on the urinary creatinine excretion [50]. Fourth, we lack information about the clinical and microbiologic response, and follow-up was not continued after ICU discharge, as this was not the primary aim of the study. Clearly, a systematic clinical PK study is required to evaluate the beneficial effects of this strategy on the outcome of sepsis patients.

Finally, we did not directly measure the body weight, and inaccurate weight estimation may have occurred for some of the studied patients [51].

## Conclusions

Patients with severe sepsis and septic shock have an increased V_d _necessitating an initial dose of ≥ 25 mg/kg TBW of amikacin to reach therapeutic peak concentrations. Even this regimen resulted in serum concentrations that were too low in one third of our patients. If using DW when calculating amikacin dose, an even higher dose should probably be considered to achieve adequate peak concentrations. The large interindividual PK variability and high amikacin concentrations at 24 hours in patients with renal impairment support the need for monitoring of serum amikacin concentrations in sepsis patients, to optimize peak concentrations, and to prevent, by increasing the dose interval, the potential toxicity of persistently high serum concentrations. It would seem important to evaluate whether this strategy could be beneficial in terms of clinical efficacy and toxicity in the sepsis population.

## Key messages

• A loading dose of ≥ 25 mg/kg TBW of amikacin is necessary to optimize peak concentrations and increase the bactericidal activity of the drug in patients with severe sepsis and septic shock

• An even higher dosage may be necessary if amikacin regimen is calculated by using DW or in underweight patients (BMI <20) to avoid underdosage

• Therapeutic drug monitoring is mandatory, as no clinical or biologic variable can predict amikacin pharmacokinetics in this population

## Abbreviations

APACHE: Acute Physiology and Chronic Health Evaluation; AUC: area under the curve; BMI: body mass index; CL: clearance; CrCl: creatinine clearance; CRP: C-reactive protein; DW: corrected body weight; IBW: ideal body weight; MIC: minimum inhibitory concentration; PK: pharmacokinetic; SOFA: sequential organ failure assessment; TBW: total body weight; V_d_: volume of distribution; V_ss_: total volume of distribution.

## Competing interests

The authors declare that they have no competing interests.

## Authors' contributions

FJ conceived the study protocol. FST, FJ, PFL, TD, and HS participated in the design and coordination of the study. FST, JLV, and FJ drafted the present manuscript. All authors read and approved the final manuscript.
